# The Soluble Epoxide Hydrolase Inhibitor AR9281 Decreases Blood Pressure, Ameliorates Renal Injury and Improves Vascular Function in Hypertension

**DOI:** 10.3390/ph2030217

**Published:** 2009-12-18

**Authors:** John D. Imig, Margaret A. Carpenter, Sean Shaw

**Affiliations:** 1 Department of Pharmacology & Toxicology and Cardiovascular Research Center, Medical College of Wisconsin, Milwaukee, WI 53226, USA; Email: macarpen@mcw.edu (M.A.C.); 2 Department of Pharmacology & Toxicology, Medical College of Wisconsin, Milwaukee, WI 53226, USA; Email: seshaw@mcw.edu (S.S.)

**Keywords:** soluble epoxide hydrolase, eicosanoids, vascular, hypertension, kidney disease

## Abstract

Soluble epoxide hydrolase inhibitors (sEHIs) are demonstrating promise as potential pharmaceutical agents for the treatment of cardiovascular disease, diabetes, inflammation, and kidney disease. The present study determined the ability of a first-in-class sEHI, AR9281, to decrease blood pressure, improve vascular function, and decrease renal inflammation and injury in angiotensin hypertension. Rats were infused with angiotensin and AR9281 was given orally during the 14-day infusion period. Systolic blood pressure averaged 180 ± 5 mmHg in vehicle treated and AR9281 treatment significantly lowered blood pressure to 142 ± 7 mmHg in angiotensin hypertension. Histological analysis demonstrated decreased injury to the juxtamedullary glomeruli. Renal expression of inflammatory genes was increased in angiotensin hypertension and two weeks of AR9281 treatment decreased this index of renal inflammation. Vascular function in angiotensin hypertension was also improved by AR9281 treatment. Decreased afferent arteriolar and mesenteric resistance endothelial dependent dilator responses were ameliorated by AR9281 treatment of angiotensin hypertensive rats. These data demonstrate that the first-in-class sEHI, AR9281, lowers blood pressure, improves vascular function and reduces renal damage in angiotensin hypertension.

## 1. Introduction

Epoxyeicosatrienoic acids (EETs) are arachidonic acid metabolites generated by cytochrome P450 epoxygenase enzymes. Soluble epoxide hydrolase (sEH) can further metabolize EETs to form dihydroxyeicosatrienoic acids (DHETs). Metabolism of EETs to DHETs is the primary mechanism whereby the biological actions of EETs are decreased or eliminated [1,2,3,4]. EETs are now recognized as major regulators of cardiovascular and renal function and increasing EET levels protects the renal and cardiovascular systems [3,4]. In the past decade, sEHIs have been developed to enhance the renal and cardiovascular protective actions offered by EETs. Previous studies have demonstrated antihypertensive and renal protective properties for sEHIs [5,6,7,8,9]. Improvements in vascular function and anti-inflammatory actions for sEH inhibitors have also been demonstrated in a number of cardiovascular disease states [7,10,11,12,13]. The present study was conducted to test the ability of a first-in-class sEHI, AR9281 to decrease blood pressure and provide renal vascular protection in a rat model of angiotensin dependent hypertension. 

## 2. Results and Discussion

### 2.1. Blood Pressure

**Figure 1:**
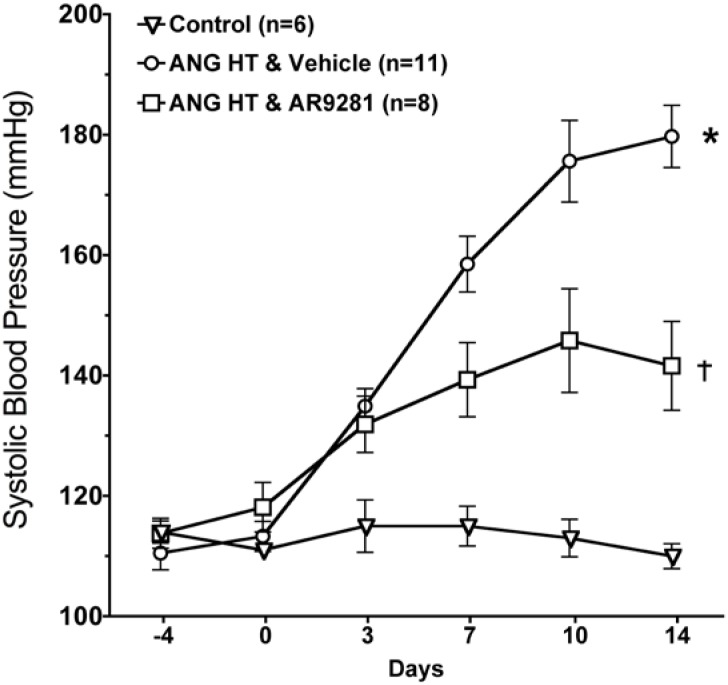
The effect of the sEHI, AR9281 on systolic blood pressures in angiotensin-infused rats (ANG HT). Values are mean ± SEM. *Significant difference between control and ANG HT & vehicle groups, =significant difference between ANG HT and vehicle and ANG HT and AR9281 groups.

The effect of the sEHI, AR9281 on blood pressure in angiotensin hypertension is shown in Figure 1. AR9281 decreased blood pressure when administered to angiotensin infused hypertensive rats. The decrease in blood pressure was evident at one week and blood pressure was maintained at a lower level throughout the second week. Blood pressure at the end of the two week treatment period averaged 110 ± 2 mmHg in controls, 180 ± 5 mmHg in angiotensin hypertension, and 142 ± 7 mmHg in angiotensin hypertension treated with AR9281. These findings are in agreement with previous studies in angiotensin hypertension that demonstrated a blood pressure lowering effect of sEHIs [7,8,9]. The mechanism for lowering blood pressure appears to be dependent on decreasing vascular resistance and increasing urinary sodium excretion [6,7,9]. These changes in vascular resistance and sodium excretion are in line with the renal and vascular actions attributed to EETs. The ability of sEHIs to lower blood pressure in rodent models of hypertension is controversial. Antihypertensive effects of sEHIs have also been demonstrated in other animal models of hypertension including deoxycorticosterone acetate (DOCA) salt hypertension [5,14,15]. On the other hand, treatment with a sEHI does not decrease blood pressure in spontaneously hypertensive rat (SHR), the stroke-prone SHR, or L-NAME induced hypertension [13,16,17]. Therefore sEHIs demonstrate antihypertensive effects in angiotensin dependent hypertension and this effect on blood pressure is observed in other but not all animal models of hypertension. 

### 2.2. Glomerular Injury

**Figure 2:**
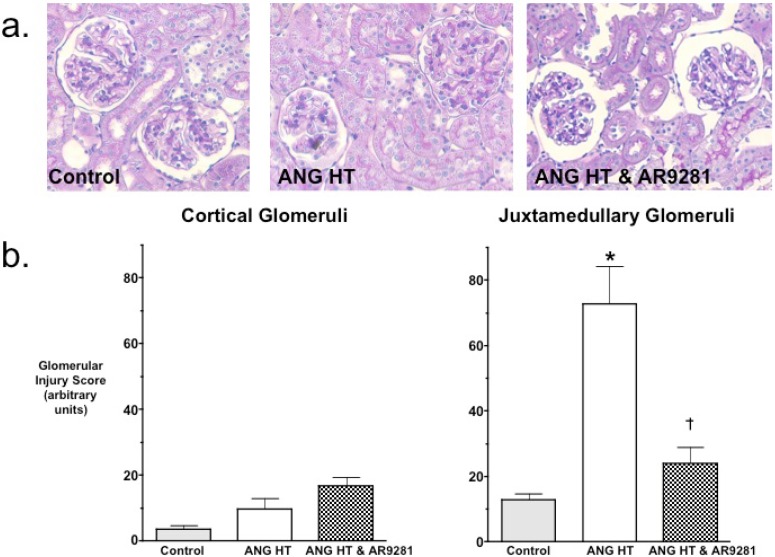
The effect of the sEHI, AR9281 on glomerular injury in angiotensin-infused rats (ANG HT). (a) Representative pictures of glomerular histological analysis. (b) Glomerular injury scores in cortical glomeruli (left graph) and juxtamedullary glomeruli (right graph). Values are mean ± SEM. *Significant difference between control and ANG HT & vehicle groups, =significant difference between ANG HT & vehicle and ANG HT & AR9281 groups.

Even though the ability for sEHIs to lower blood pressure have been variable, the ability of sEHIs to protect from end organ damage in hypertension has been much more consistent. Experimental findings have demonstrated that the glomeruli in the juxtamedullary region of the kidney cortex are the first to be injured in hypertension [18]. Others and we have shown previously that two-weeks of angiotensin dependent hypertension results in mild glomerular injury [7,9,18]. Therefore we evaluated cortical and juxtamedullary glomeruli for injury in the animal groups. Histological evaluation of glomerular injury revealed that AR9281 treatment decreased injury to the juxtamedullary glomeruli (Figure 2). Glomerular injury increased 6-fold in juxtamedullary glomeruli of angiotensin hypertensive rats and was elevated by only 2-fold in angiotensin hypertensive rats treated with AR9281. The decrease in glomerular injury observed in the present experimental studies is in accord with previous findings in angiotensin hypertension [7,9]. This decrease in renal injury could be in part due to the decrease in blood pressure; however, there is now strong evidence that sEHIs can decrease renal injury as well as heart and brain injury independent of lowering blood pressure [12,13,19,20,21,22]. Thus the findings of the present study demonstrate that AR9821 can decrease renal injury associated with angiotensin hypertension. 

### 2.3. Renal Inflammation

Comparing mRNA expression of inflammatory genes between groups assessed renal inflammation. Real-time PCR arrays were used to profile mRNA expression of 84 inflammatory cytokines and receptors in the kidney cortex. Data presented in Table 1 demonstrate that two weeks of angiotensin hypertension increased the expression of 30 inflammatory genes greater than 3-fold. AR9281 treated angiotensin hypertensive rats had a 3-fold or greater decrease in 34 renal inflammatory genes when compared to vehicle treated angiotensin hypertensive rats. There is growing evidence that hypertension is an inflammatory disease and that anti-inflammatory treatments can slow the progression of hypertension and renal injury [23,24,25,26,27]. This finding agrees with previous studies from others and we that demonstrated increased macrophage infiltration into the kidney of angiotensin hypertension [7,9,18]. More recent studies have implicated a role for T cells in the genesis of angiotensin dependent hypertension [28,29]. Interestingly, the data in the present study point to T-cell activation in angiotensin dependent hypertension and T cell inactivation by the sEHI AR9821. The chemokine ligand CCl25 and its receptor Ccr9 increased in hypertension and were decreased by AR9821. Three interleukin family genes linked to T-cells, IL-3, IL-4 and IL-5 changed in a similar manner. Two other chemokines, Ccl11 and Ccl22 and their respective receptors Ccr3 and Ccr4 were increased in hypertension and decreased by AR9821. The results of this PCR array indicates that AR9821 decreased mRNA expression of inflammatory cytokines in angiotensin hypertension which supports the notion that AR9821 therapy provides renal protection via anti-inflammatory effects.

**Table 1:** Renal inflammatory gene expression changes in angiotensin-infused rats (ANG HT). Renal proinflammatory mRNA expression increased in ANG HT and AR9821 decreased proinflammatory gene expression in ANG HT. Grey shading highlights genes increased greater than 3-fold by hypertension and decreased 3-fold by AR9821.

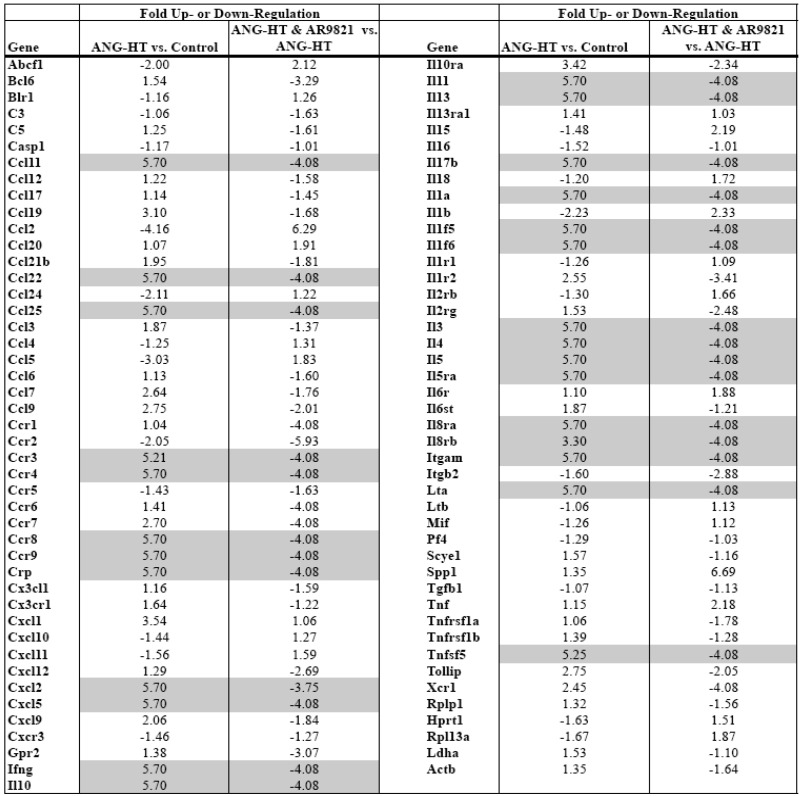

### 2.4. Renal and Mesenteric Vascular Function

**Figure 3:**
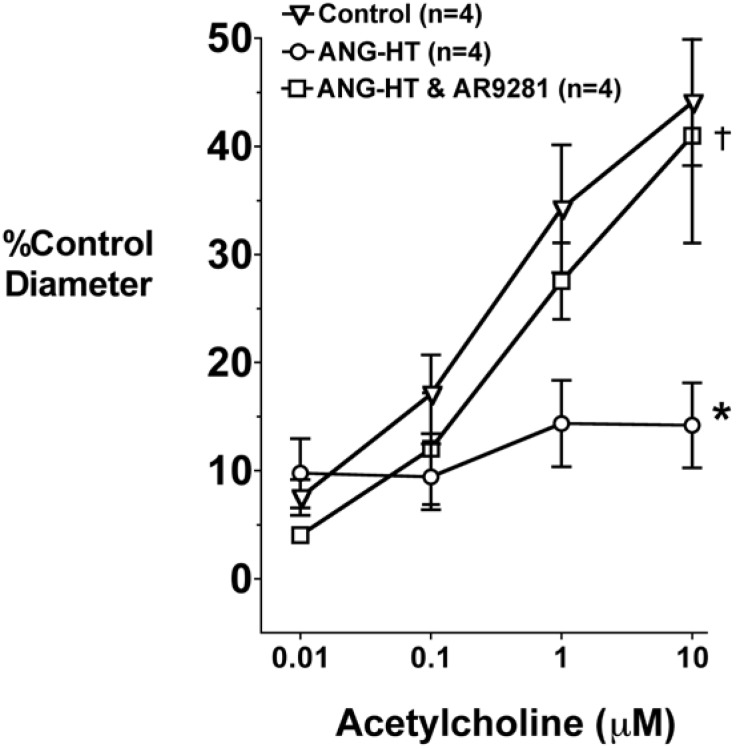
The effect of the sEHI, AR9281 on afferent arteriolar vasodilation in angiotensin-infused rats (ANG HT). Values are mean ± SEM. *Significant difference between control and ANG HT and vehicle groups, =significant difference between ANG HT & vehicle and ANG HT and AR9281 groups.

Afferent arteriolar dilator responses to acetylcholine in the animal groups are presented in Figure 3. Acetylcholine dose-dependently increased afferent arteriolar diameter in control rats. The afferent arteriolar dilation to acetylcholine was significantly blunted in angiotensin dependent hypertension. Afferent arteriolar dilation averaged 44 ± 6% in control animals and 14 ± 4% in angiotensin hypertension in response to 10 μM acetylcholine. This attenuation in acetylcholine mediated dilation is similar to that previously reported by our group [7,30,31]. Treatment with AR9281 restored the afferent arteriolar dilator response in angiotensin hypertension and diameter increased by 41 ± 9% to 10 μM acetylcholine.

Flow-mediated dilation in mesenteric resistance arteries was also assessed. An increase from zero flow to 50 μL/min (low flow) or zero to 300 μL/min (high flow) in mesenteric resistance arteries resulted in a 20 ± 4% and 49 ± 4% (n = 4) relaxation, respectively in control rats. This vasodilatory response was blunted in angiotensin hypertensive rats and averaged 14 ± 1% to low flow and 20 ± 1% (n = 3) to high flow. AR9281 treatment restored the mesenteric resistance arteries responses to low flow (21 ± 9%) and high flow (48 ± 1%; n = 3) in angiotensin dependent hypertensive rats. 

The findings of impaired endothelial mediated responses in renal and mesenteric arterioles is in agreement with numerous studies that have demonstrated endothelial dysfunction in animal models of hypertension and human hypertension [30,31,32,33]. Improved endothelial responses in patients with cardiovascular disease have been associated with decreased cardiovascular events and mortality. Therefore the fact that AR9821 treatment improved endothelial function in angiotensin hypertension suggests that this sEHI would have beneficial effects for the overall health of patients with cardiovascular disease.

## 3. Experimental Section

### 3.1. Animals

The Medical College of Wisconsin Institutional Review Committee according to National Institutes of Health Guidelines approved all animal studies for the Care and Use of Laboratory Animals. Male Sprague-Dawley rats were divided into three experimental groups; the first group was subjected to sham surgery, the second group received angiotensin and vehicle treatment (5% (2-hydroxypropyl)-β-cyclodextrin), and the third group received angiotensin and AR9281 (100 mg/Kg/d, Arête Therapeutics, Hayward, CA, USA), a SEH transition state inhibitor of the 1,3-disubstituted urea, carbamate and amide class. The dose of AR9281 was based on its demonstrated ability to inhibit sEH *in vitro* and *in vivo* and known pharmacokinetic profile in rats. Angiotensin was infused at a continuous rate via a mini-pump (65 ng/min). Systolic blood pressure was measured using tail-cuff plethysmography.

### 3.2. Evaluation of Glomerular Injury

At the end of the two week AR9281 treatment period, kidneys were immediately fixed in 10% buffered formalin solution and embedded in paraffin for light microscopic evaluation. Sections were cut at a thickness of 2 to 3 μm and stained with hematoxylin-eosin, periodic acid-Schiff reagent and periodic acid-methenamine-silver. For semiquantitative evaluation, two individuals evaluated histological sections for renal injury in a blind fashion. Approximately 30 subcapsular and 30 juxtamedullary glomeruli from each specimen were analyzed for glomerular injury: Grade 1, normal glomerulus by light microscopy; Grade 2, involvement of up to one-third of the glomerular area; Grade 3, involvement of one to two thirds of the glomerulus; and Grade 4, two-thirds to global sclerosis. Histological sections were evaluated from four animals in each group and an average score for each category determined. 

### 3.3. Real-Time Polymerase Chain Reaction (PCR) Array Gene Expression Profiling

Total RNA was extracted from 20 mg kidney cortex using the RNeasy^®^ Plus Mini kit (Qiagen, Valencia, CA, USA) according to the manufacturer’s protocol. RNA concentrations were determined using absorbance at 260nm. Reverse-transcription was performed on 2μg of RNA from each sample using the RT^2^ PCR Array First Strand Kit (SuperArray Biosciences, Frederick, MD, USA). Each cDNA synthesis reaction was diluted before being added to an RT^2^ Real-Time SYBR Green PCR Mastermix (SupoerArray) which was aliquoted onto a 96-well PCR Array plate, one sample per plate; each well contained a primer pair for a different gene or control. Thermal cycling and real-time detection were done with a Bio-Rad iCycler (Bio-Rad Laboratories, Hercules, CA, USA): step 1) 95 °C for 10 minutes, step 2) 95 °C for 15 seconds followed by 60 °C for 60 seconds (repeated 40 times). Melt-curve analysis was completed after each PCR reaction. Analysis was conducted using templates provided by SuperArray Biosciences.Threshold cycle (C_t_) values were normalized to a set of housekeeping genes Rplp1, Hprt1, Rpl13a, Ldha, and Actb as recommended by SuperArray Biosciences to get a ΔC_t_ value and fold-changes were calculated using the equation: (2^-ΔCt^ test)·(2^-^^ΔCt ^control)^-1^. 

### 3.4. In Vitro Perfused Juxtamedullary Nephron Experiments

Rats were anesthetized with pentobarbital (40 mg/kg body weight i.p.). The right kidney was isolated and after a midline laparotomy, the right renal artery was cannulated through the superior mesenteric artery. The kidney was immediately perfused with a Tyrode’s solution containing 6% albumin and a mixture of L-amino acids. After the microdissection procedures were completed, the renal artery perfusion pressure was set to 100 mm Hg. The tissue surface was continuously superfused with a Tyrode’s solution containing 1% albumin. After a 20-minute equilibration period, an afferent arteriole was chosen for study, and baseline diameter was measured. After the control period, the afferent arteriole was constricted with phenylephrine and the endothelium-dependent relaxation was assessed using increasing concentrations of acetylcholine (0.01–10 μm). The afferent arteriole diameter changes to acetylcholine were monitored for 3 minutes at each concentration. Steady-state diameter to acetylcholine was attained by the end of the second minute, and the average diameter at the third minute was used for statistical analysis. 

### 3.5. Mesenteric Resistance Artery Diameter responses

Mesenteric artery segments were obtained from the rats and mounted between two cannulae in a pressure myograph system (Danish Myo Technology model 111P). The interior and exterior of the vessel were in oxygenated (95% O2/5% CO2) Krebs physiological salt solution (PSS, mmol/L:119.0 NaCl, 25.0 NaHCO_3_, 4.6 KCl, 1.2 KH_2_PO_4_, 1.2 MgSO_4_, 1.8 CaCl_2_, 11.0 glucose, Sigma) at pH 7.4 and 37 ºC. Under no flow conditions, over a span of 18 min, the pressure within the vessel was increased at 10 mmHg increments from 20 to 65 mmHg. The vessel was then equilibrated at 65 mmHg for 30 min and remained at that pressure for the duration of the experiment. Lumen diameter measurements were acquired and logged using the MyoView 1.2P user interface. The control lumen diameter was calculated as the mean diameter during the last 15 min of the 30 min equilibration. Diameter of the constricted vessel was calculated as the mean during the last 2 min of 15 min following the addition of U46619. Following U46619 treatment, the mesenteric artery diameter responses to low (50 μL/min) or high flow (300 μL/min) were determined.

### 3.6. Statistics

Data presented are mean ± SEM. The significance of differences between groups for the blood pressure and renal vascular data were evaluated with an analysis of variance (ANOVA) for repeated measures followed by a Duncan’s multiple range post hoc tests. An unpaired t-test was applied to compare the histological grading. A *P* value of <0.05 was considered significant. 

## 4. Conclusions

The findings of the present study demonstrate the anti-hypertensive effect of AR9281 in an angiotensin dependent model of hypertension. In addition to decreasing blood pressure, AR9281 decreased glomerular injury and renal inflammation. Endothelial dilator responses of the afferent arterioles and mesenteric resistance arteries were also improved in angiotensin hypertensive rats receiving AR9281. More importantly, these results provide strong evidence that besides lowering blood pressure the first-in-class sEHI, AR9821 has added renal and cardiovascular beneficial effects in angiotensin dependent hypertension. The additional anti-inflammatory actions, vascular endothelial and end organ protection provided by AR9281 makes this a promising pharmaceutical agent for the treatment of cardiovascular disease, diabetes, inflammation, and kidney disease. 
